# Supplementary material for: Automatic precursor recognition and real-time forecasting of sudden explosive volcanic eruptions at Whakaari, New Zealand
Source: Nat Commun. 2020 Jul 16;11:3562. doi: 10.1038/s41467-020-17375-2 (PMC7367339; doi:10.1038/s41467-020-17375-2)
Supplement: Supplementary file 1 — Supplementary Information [file 41467_2020_17375_MOESM1_ESM.pdf]

Supplementary Information for

Automatic precursor recognition and real-time forecasting of sudden explosive volcanic eruptions at Whakaari, New Zealand

by Dempsey et al.

Supplementary Figures

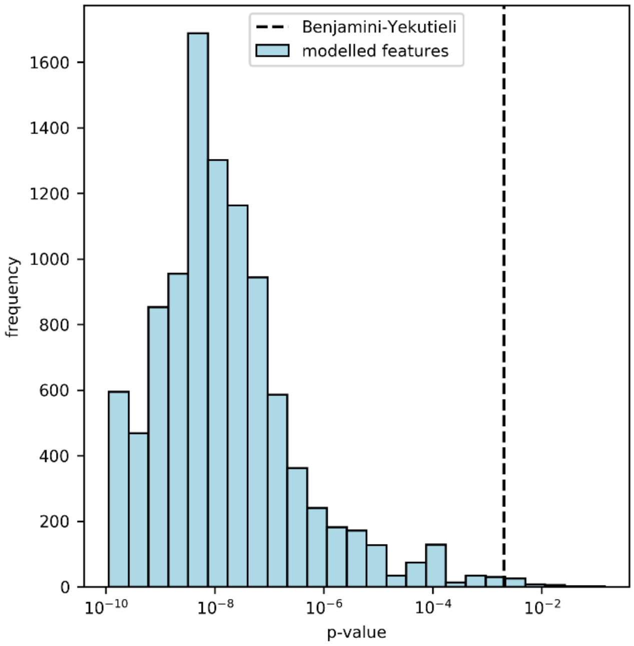

Supplementary Figure 1: Frequency of features used in classification modelling by p-value. A Benjamini-Yekutieli cut-off for a false discovery rate of 5% is indicated by the dashed line.

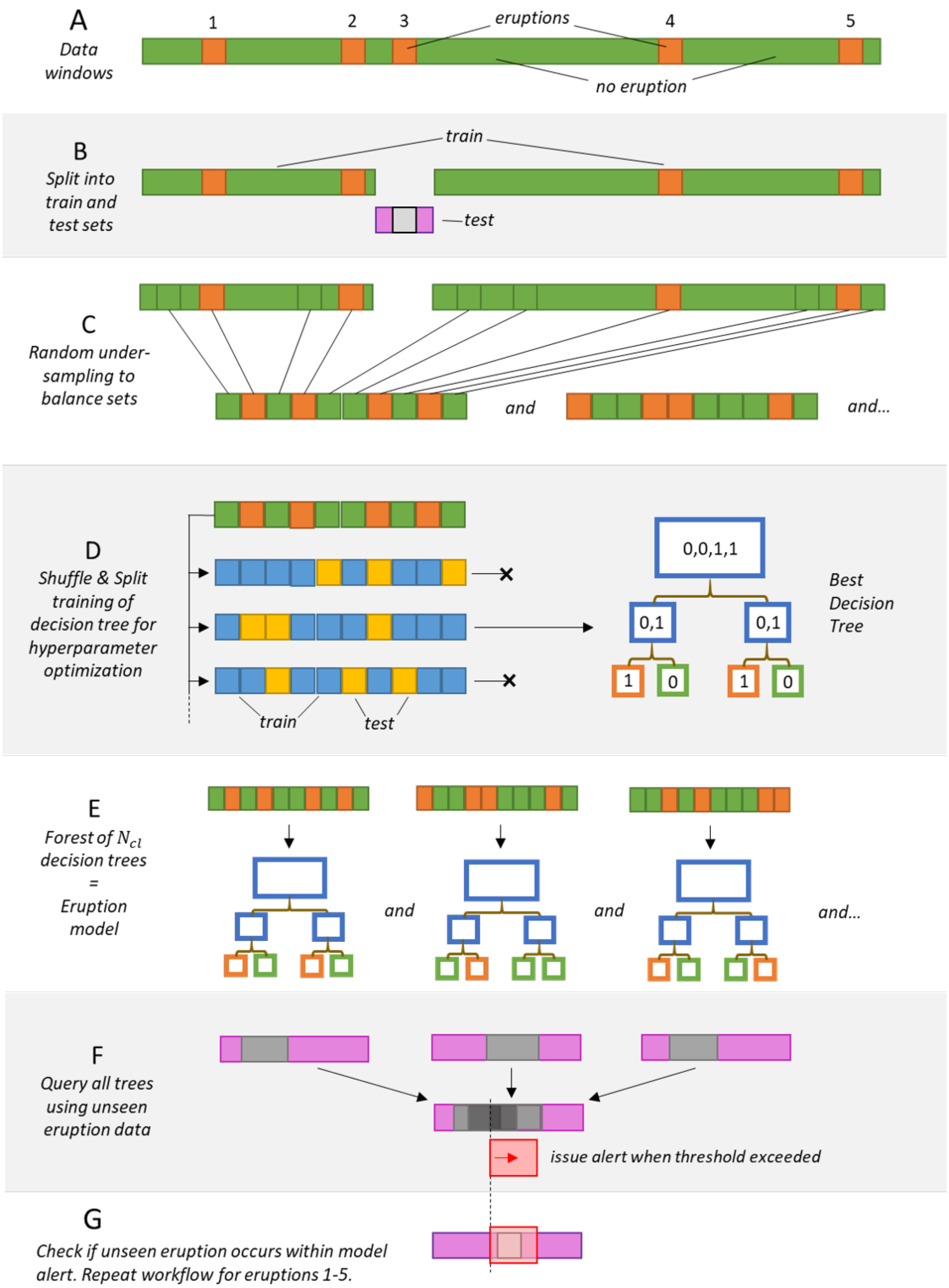

Supplementary Figure 2: Cross validation model workflow. A. Classifying eruptive (orange) and non-eruptive windows. B. Splitting a test eruption (grey/purple) from the training data (orange/green). C. Undersampling the non-eruptive windows to create multiple balanced datasets. D. For each undersampled dataset, use Shuffle & Split to train a decision tree. E. Eruption model as a Random Forest of decision trees specialized on eruptive windows. F. Testing the eruption model on unseen data and issuing alerts at sufficient agreement. G. Compare modeled alerts to unseen eruption. Repeat steps B-G for other four eruptions.

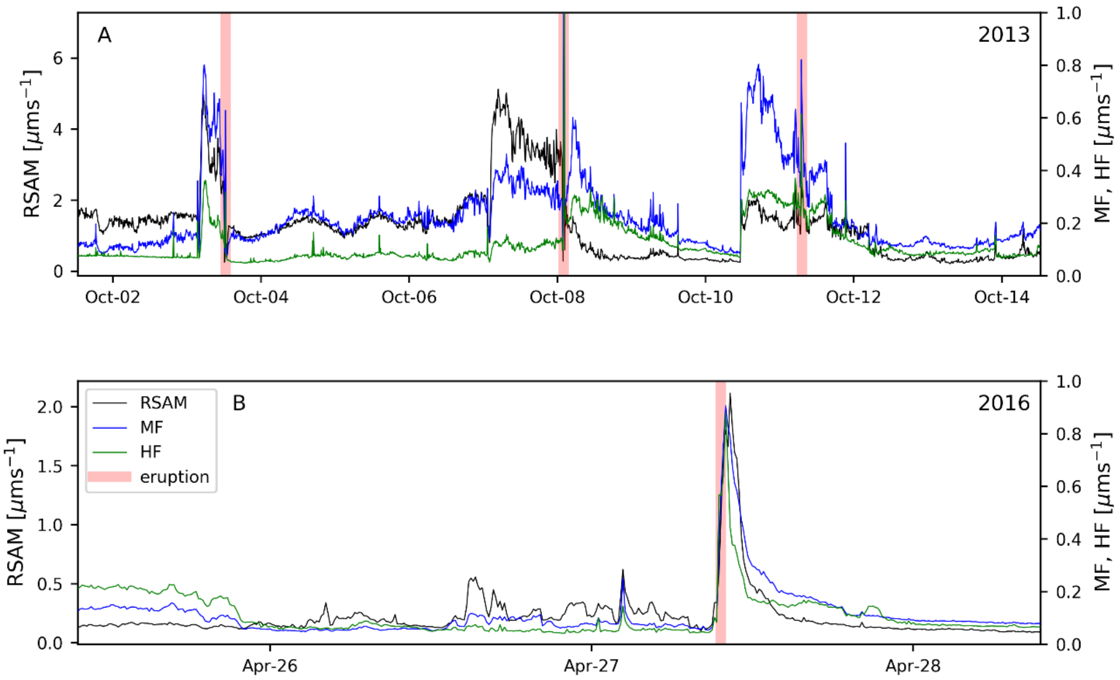

Supplementary Figure 3: RSAM (black), MF (blue) and HF (green) data streams for two missed eruptions. The timing of eruption are indicated by the vertical red bars. A. The 8 Oct 2013 eruption, the middle of three during the eruptive period. B. April 2016 eruption.

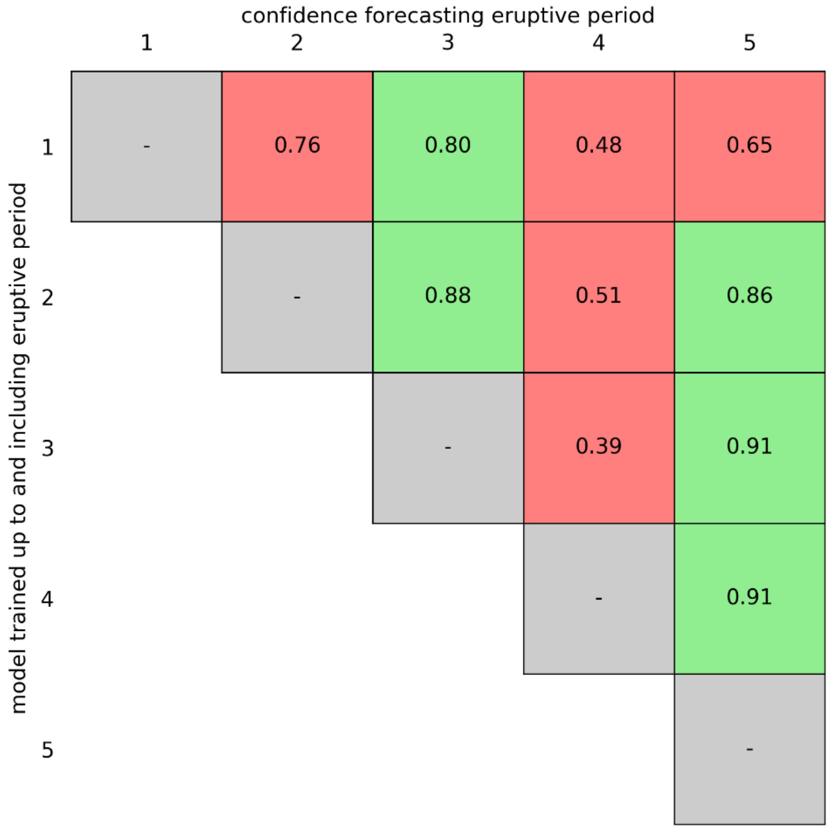

Supplementary Figure 4: Matrix of retrospective forecasting outcomes. Rows indicate forecast models trained using consecutive eruptive periods, e.g., row two is trained using the 2012 and Aug 2013 eruptions and data up to one month before the Oct 2013 eruptive period. Columns indicate the performance of a forecaster on a future eruptive period as measured by model confidence, which is the maximum ensemble mean in the 48 hours prior to the eruptive period. For example, row three, column five indicates that a forecast model trained using the 2012 and two 2013 eruptive periods will anticipate the 2019 eruption with a confidence of 0.91.

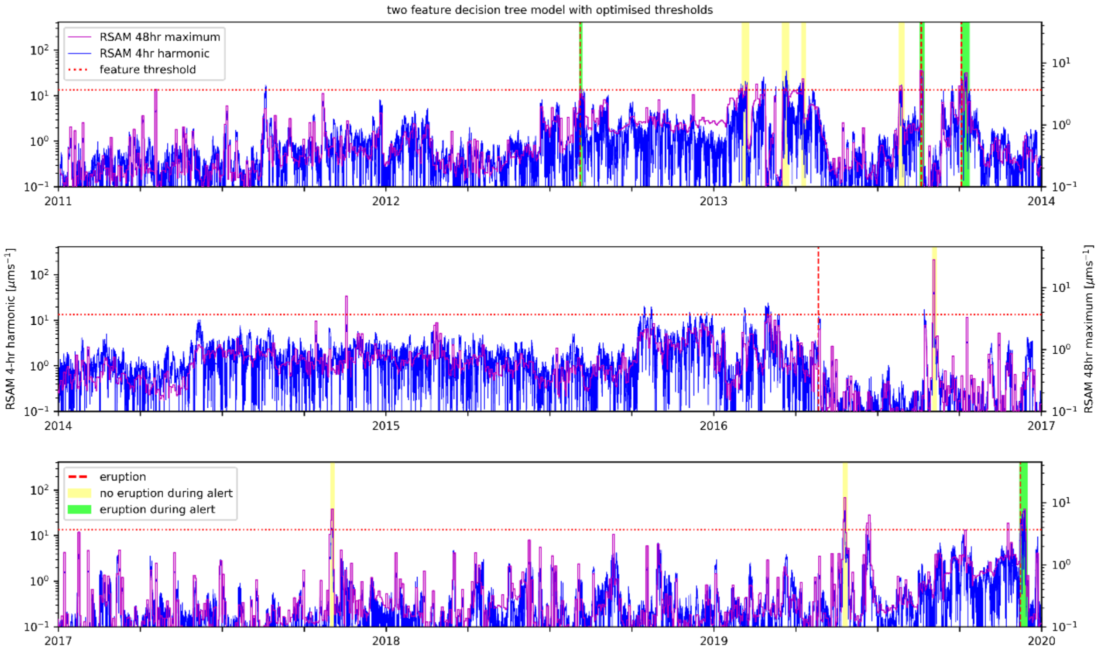

Supplementary Figure 5: Alternative parameter set for the forecast model illustrating potential overfitting. Restricted to two user-selected features – RSAM maximum (magenta) and 4 hr harmonic (blue) – and a two node decision tree with user-selected thresholds that were optimised using knowledge of the entire dataset. The forecast model generates twelve alert periods that cover six eruptions, missing only the Apr 2016 event, and the total in alert duration is 1.7% of the analysis period (56 days).

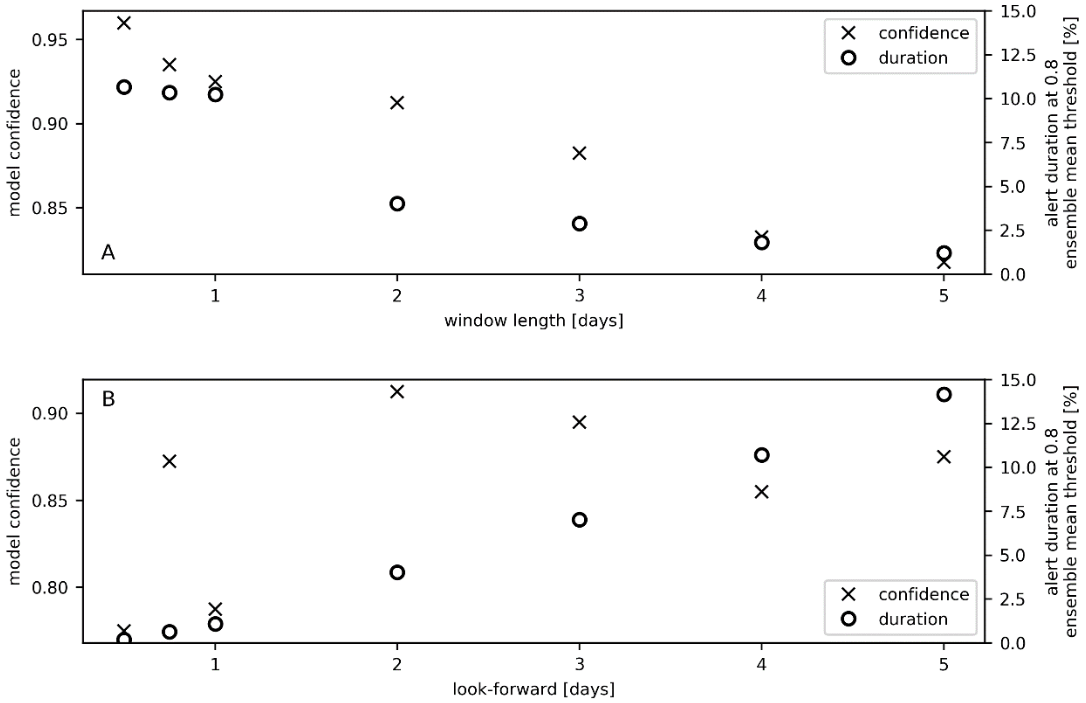

Supplementary Figure 6: A. Forecast model performance versus window length, T_w, expressed as confidence ahead of the Dec 2019 eruption (crosses) and total alert duration (circles). B. Forecast model performances versus look-forward period, T_{lf}.

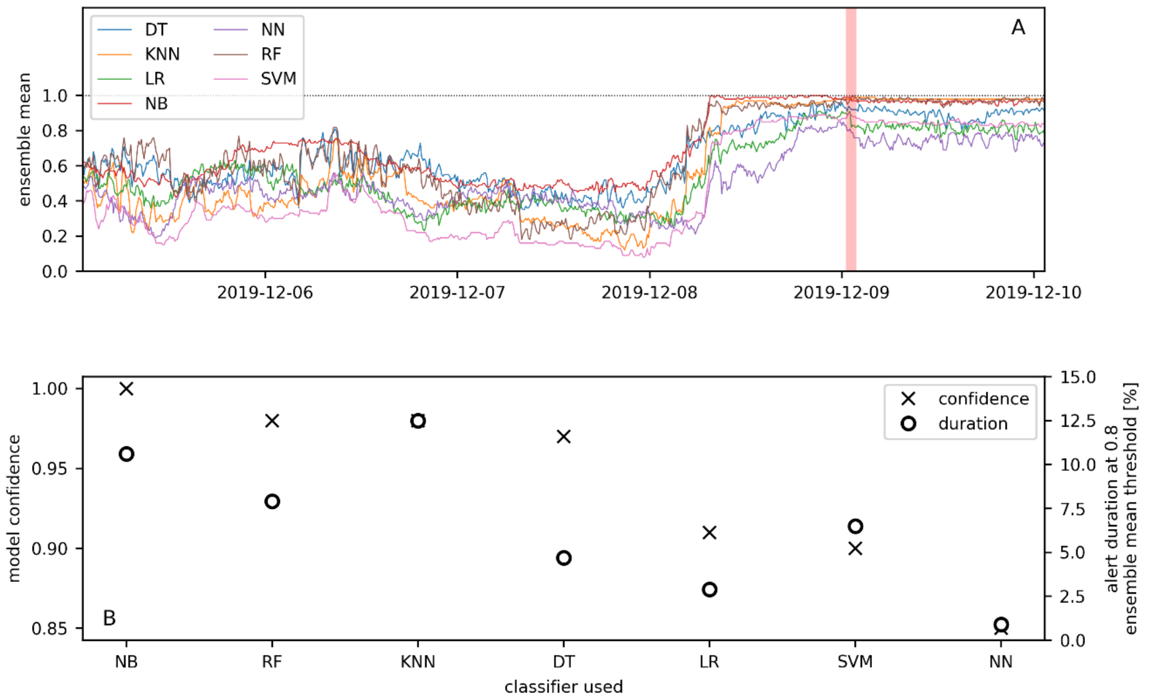

Fig 7: A. Ensemble mean from a forecast model in the period preceding the Dec 2019 eruption, for seven different classifier models: DT = Decision Tree, KNN = k-Nearest Neighbors, LR = Logistic Regression, NB = Gaussian Naive Bayes, NN = Neural Network, RF = Random Forest, SVM = Support Vector Machine. B. Performance of the seven forecast models, in terms of confidence of imminent eruption (crosses) and total alert duration (circles). Total alert duration increases with the number of issued alerts and therefore is a proxy for the false positive rate.

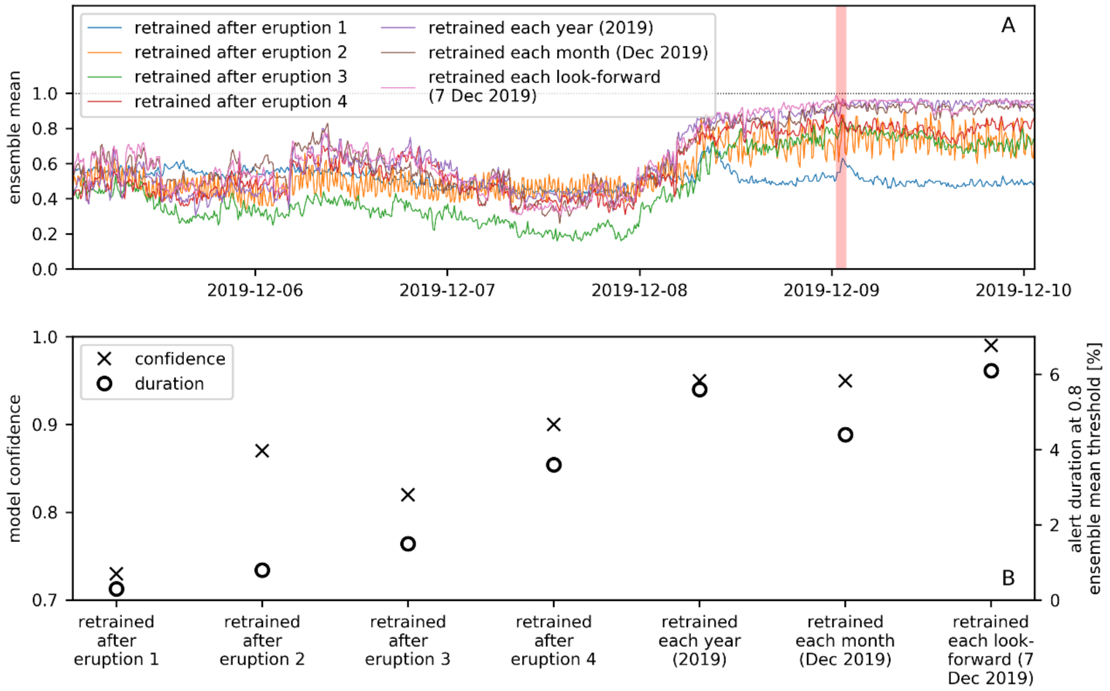

Supplementary Figure 8: A. Ensemble mean from a forecast model in the period preceding the Dec 2019 eruption for seven different training intervals: from Jan 2011 up to one month after each of the first four eruptions, and after the first four eruptions plus retraining every year, month or look-forward period. B. Performance of the seven forecast models, in terms of confidence of imminent eruption (crosses) and total alert duration (circles).

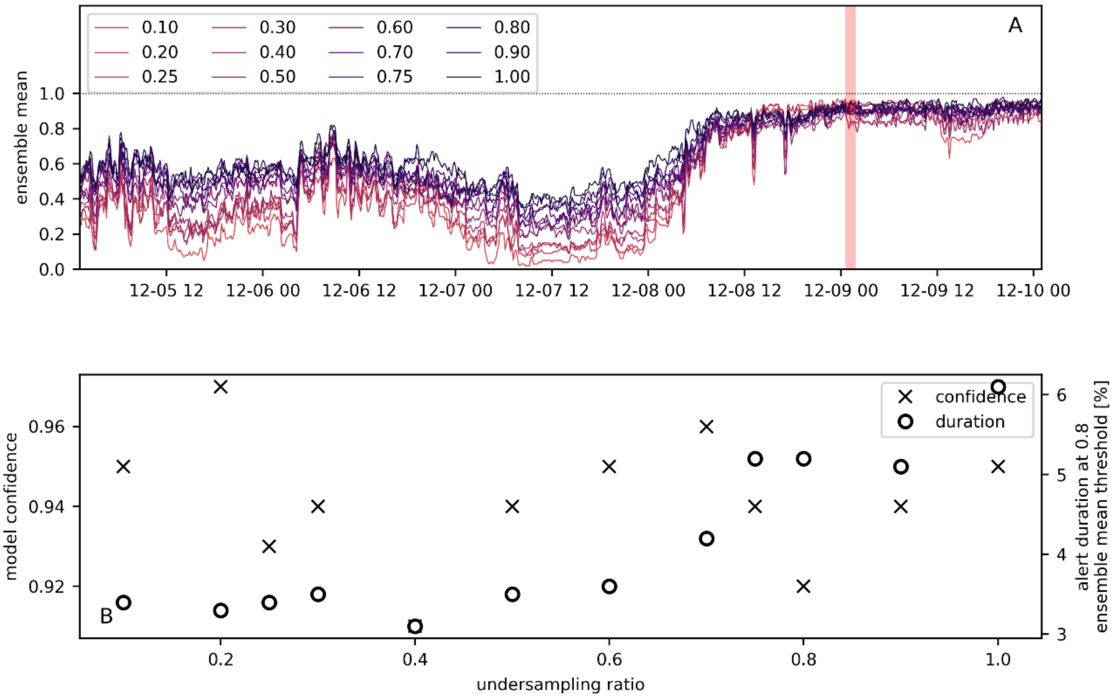

Supplementary Figure 9: A. Ensemble mean from a forecast model in the period preceding the Dec 2019 eruption, for different values of the undersampling ratio. B. Performance of the different forecast models, in terms of confidence of imminent eruption (crosses) and total alert duration (circles).

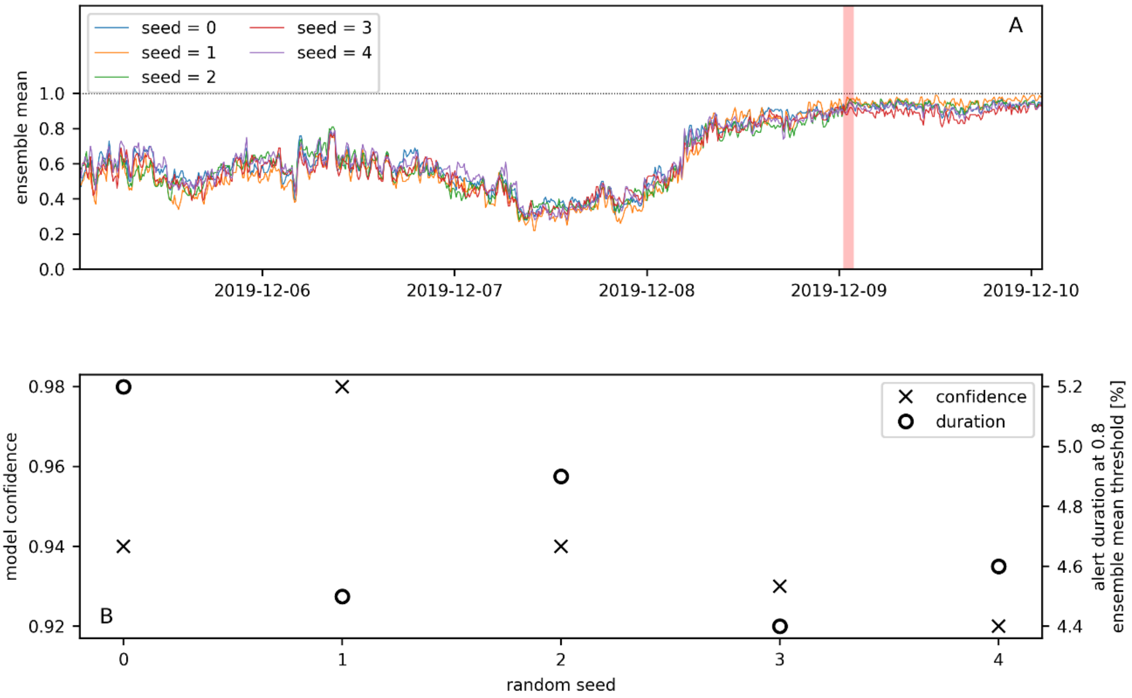

Supplementary Figure 10: A. Ensemble mean from a forecast model in the period preceding the Dec 2019 eruption, for five consecutive integer seeds of the random number generator. B. Performance of the five forecast models, in terms of confidence of imminent eruption (crosses) and total alert duration (circles).

Supplementary Tables

Supplementary Table 1: Summary of Volcano Alert Bulletins for Whakaari issued by GNS Science, available at https://www.geonet.org.nz/volcano/vab/. Eruption reports highlighted. Note, prior to July 2014, New Zealand operated a VAL (volcano alert level) system with one unrest level (VAL 1) and four categories of eruption (VAL 2-5). After July 2014, this was replaced with by a scheme with two unrest levels (VAL 1-2) and three eruptive levels (VAL 3-5).

Date (UTC)	ID	VAL	Comment
10/23/2008 0:00	2008/04	1	Crater lake refills after Oct 2007, 15 m deep, 9 m below outfall. Steam/gas emission and acid rain.
8/2/2012 3:00	2012/01	1	Lake re-filling after 2011. 27-28 July sudden rise of 3-5 m. SO_2 gas increasing and tremor. Crater floor now rising.
8/6/2012 5:30	2012/02	2	Small eruption on 5/8/2012 at 04:55 (local time) from Crater Lake. Tremor on 4-5/8 ended in EQ at 04:54.
8/8/2012 4:30	2012/03	2	2330 on 7/8 tremor decreased. Ash entered a steam-dominated plume. Small cone forming in lake.
8/8/2012 12:00	2012/05	2	First ash since 2001. Ash plume up to 300 m. Tuff cone and ballistic impacts. Two craters within crater lake.
8/9/2012 19:30	2012/07	2	Variable ash content in plume.
8/10/2012 0:00	2012/06	2	Low tremor. Weak ash plume a few 100 m high.
8/11/2012 19:30	2012/08	2	Low altitude plume of steam and gas. Low tremor. Intermittent minor ash.
8/11/2012 22:00	2012/14	1	Ash declined. Seismicity low. SO_2 normal.
8/12/2012 19:30	2012/09	2	Weak tremor. Minor ash.
8/13/2012 0:00	2012/10	2	Low tremor. Weak ash plume a 100-300 m high. Lower gas levels.
8/13/2012 18:30	2012/11	2	Low seismicity. Weak ash plume a 100-300 m high.
8/14/2012 19:00	2012/12	2	Minor steam plume.
8/15/2012 19:00	2012/13	2	Minor steam plume.
8/27/2012 3:00	2012/15	1	Airborne gas samples 1, 10, and 17/8/2012 in typical limits. Fumaroles typical temperature.
12/12/2012 1:30	2012/16	2	Lava dome extruded in August in crater at base of cliff/edge crater, 20-30 m diameter, possibly 2 weeks or longer ago. First ever seen.
12/20/2012 1:00	2012/17	2	Dome not growing.
1/7/2013 2:00	2013/01	1	No dome growth. Normal gas. Elevated tremor. Glow at night.
1/21/2013 22:00	2013/02	1	Hydrothermal/gas jetting (mud geysering) from small hot lake, since late 2012, now semi-continuous. Elevated tremor.
1/24/2013 4:00	2013/03	1	Tremor decreased. Hybrid earthquakes appear.
1/25/2013 5:00	2013/04	1	Gas normal. Vigorous mud geysering from crater lake.
1/29/2013 1:00	2013/05	1	Continuous tremor switched to intermittent tremor. Crater lake drying out. Geysering bursts ejecting ash and ballistics. Steam/ash plumes visible from mainland.
1/29/2013 23:30	2013/06	1	Lake dried, tuff cone forming. Bursts of ash/mud/rock 50-100 m high.
1/31/2013 5:00	2013/07	1	Gas slightly higher than normal. Tremor continuous. Small explosions.
2/11/2013 1:30	2013/08	1	Tremor dropped by half. Small explosions became smaller. Lake re-forming.

2/23/2013 3:30	2013/09	2	Minor ash venting 11:30-13:30.
2/25/2013 4:00	2013/10	2	Ash emission ceased. Tremor increased.
3/4/2013 4:00	2013/11	1	Ash emission ceased. Small cone built in the hot lake.
3/26/2013 3:00	2013/12	1	Passive steaming and degassing with low tremor alternating with minor mud and steam explosions during stronger tremor (<1 day to over 1 week).
4/29/2013 1:00	2013/13	1	No small mud/ash eruptions for 1 month. Low tremor and gas.
7/26/2013 6:00	2013/14	1	Minor activity over last 24 hrs, increased tremor. Explosive jets (mud geysering) through crater lake 20-30 m vertically.
8/5/2013 0:00	2013/15	1	Minor activity declined.
8/19/2013 23:10	2013/16	2	Small eruption on 20/8/2013 at 10:23 (local), 10 min long. Steam mainly produced.
8/20/2013 2:00	2013/17	2	Eruption plume 4 km high. Local mud/ballistics. Mainly steam in plume. Crater basin excavated.
8/20/2013 18:30	2013/18	2	Low level activity
8/21/2013 1:00	2013/19	1	Steam and gas emitted from active crater.
8/26/2013 3:00	2013/20	1	Gas at normal levels. Lake reforming over vents.
10/7/2013 2:00	2013/21	1	Energetic steam-venting event on 4/10/2013 at 16:36 (local). Seen from mainland. Slightly elevated tremor. Gas slightly elevated.
10/8/2013 5:30	2013/22	1	Tremor rose 15:05-15:20. Minor steam/mud eruption see from mainland.
10/12/2013 0:00	2013/23	2	Moderate explosive eruption on 11/10/13 at 20:09 (local). Ash and mud eruption for 1 minute. "…larger than recent events and would have been life threatening to people on the island."
10/14/2013 1:45	2013/24	2	Tremor decreasing.
10/21/2013 2:30	2013/25	1	Gas flux remains high.
11/3/2013 23:30	2013/26	1	Low seismic and gas levels.
12/23/2013 3:30	2013/27	1	Variable gas.
1/21/2014 21:00	2014/01	1	Gas elevated. Lake growing.
2/10/2014 23:00	2014/02	1	Low activity levels. Lake rising.
8/27/2014 23:00	2014/03	1	Small earthquakes <10 km depth. Gas normal.
10/5/2014 21:53	2014/04	1	Volcanic unrest at low levels. Last report under old VAL system.
10/12/2015 12:00	2015/01	1	Slight increase in tremor and gas since 8/10/15. Lake risen 2 m since June. First report under new VAL system.
11/2/2015 2:00	2015/02	1	Tremor and gas decreased.
12/9/2015 22:00	2015/03	1	Minor ground deformation (+20 to – 12 mm). Small gas increase. Tremor rising.
4/26/2016 23:00	2016/01	1	Lake level dropped by 2 m. Gas/tremor at typical values.
4/27/2016 23:33	2016/02	3	Eruption on 27/4/2016 at 21:50 (local). Moderate seismicity briefly.
4/28/2016 6:45	2016/03	2	Low seismic energy and no change in SO_2. Ash to 500 m away.
4/29/2016 22:45	2016/04	2	New crater formed by 27/4 eruption. No new magma. Steam-driven eruption like 2012-2013. Normal seismic and gas.
5/2/2016 1:00	2016/05	2	Gas/seismic normal.
5/9/2016 0:00	2016/06	1	Seismic/gas decreased.

5/24/2016 20:50	2016/07	1	Eruption excavated 13-15 of lake floor sediment.
9/13/2016 0:50	2016/08	3	Minor ash passively emitted from a vent on 2012 lava dome from 11:50.
9/13/2016 3:15	2016/09	3	Seismic activity low, minor.
9/13/2016 23:15	2016/10	3	Minor volcanic activity. Small amounts of passive ash emission. Seismicity/gas low.
9/15/2016 0:15	2016/11	2	No new magma in ash. Gas-venting not explosive eruption.
9/18/2016 23:00	2016/12	1	Steam plumes.
10/3/2016 3:20	2016/13	1	Gas emitted from a joint on the lava dome. Strong gas flow. Lake dropped by 3 m and mostly gone.
4/3/2017 2:00	2017/01	1	Hot clear gas continues to be emitted. No lake. Normal gas and tremor.
5/30/2018 2:15	2018/01	1	Lake forming. Gas from dome continues. Seismic and gas low. “loud sounds”
7/4/2018 1:40	2018/02	1	Lake growing. Seismic/gas low.
4/16/2019 2:00	2019/01	1	Lake peaked in January, dropped by 2 m. Steady generally low level. Tremor low. SO_2 normal.
5/25/2019 20:30	2019/02	1	Local earthquake swarm.
6/4/2019 2:30	2019/03	1	Swarm continues but decreasing intensity (356 earthquakes).
6/20/2019 2:20	2019/04	1	Earthquake swarm started at 04:00 (local) in same location as May swarm.
6/26/2019 6:30	2019/05	2	Highest gas output since 2013.
6/30/2019 23:45	2019/06	1	Nearby earthquake swarms continue at lower levels.
9/25/2019 21:30	2019/07	1	Geyser-burst eruptions in crater lake (up to 10 m). Seismic/gas minor/low.
10/30/2019 0:30	2019/08	1	Gas increasing and tremor highest since 2016.
11/17/2019 23:00	2019/09	2	Increased SO_2 and tremor. Earthquakes continuing. Geysering.
11/25/2019 1:00	2019/10	2	Gas emissions high-moderate. Tremor moderate. Geysering.
12/3/2019 0:00	2019/11	2	Gas/mud bursts (20-30 m-high). Ejecta deposited around lake. Elevated gas and seismicity.
12/9/2019 1:30	2019/12	4	Eruption at 14:11 (local).
12/9/2019 3:25	2019/13	3	Ash to 4000 m. Ash seen on island.
12/9/2019 22:40	2019/14	3	Seismic activity dropped. Local steam and mud jetting.
12/10/2019 3:20	2019/15	3	Seismic weak. Geysering continues.
12/10/2019 21:30	2019/16	3	Tremor increased since 04:00.
12/11/2019 4:30	2019/17	3	Tremor now highest since 2016. High gas output.
12/11/2019 21:20	2019/18	2	Tremor high. Steam and mud jetting from vents.
12/12/2019 4:00	2019/19	2	Tremor remains high.
12/12/2019 22:00	2019/20	2	Tremor decreased but still elevated. Vigorous geysering. Gas high and increasing.
12/13/2019 4:30	2019/21	2	Tremor declined.
12/13/2019 22:30	2019/22	2	Tremor declined. Ash emission near vent. Vent glow at night. Gas jetting.

12/14/2019 21:30	2019/23	2	Low tremor. Glow from vent. Active crater emitting gas.
12/16/2019 0:30	2019/24	2	Three main vents in a 100 m^2 area. High-temperature transparent gas from one. Other vents producing steam and gas.
12/16/2019 22:45	2019/25	2	Low tremor. All vents the same. High gas output.
12/17/2019 23:00	2019/26	2	Low tremor.
12/19/2019 0:00	2019/27	2	Tremor low. Gas/steam continues.
12/20/2019 1:00	2019/28	2	Hot gas and steam release. 12-m high landscape scarp.
12/23/2019 0:10	2019/29	2	Hot gas and steam.
1/6/2020 7:30	2020/01	2	Hot gas and steam, glow. Tremor low since 14/12/2019. Small amounts of ash on 23 and 26/12/2019, vent collapse. SO_2 returned to normal.
1/14/2020 21:15	2020/02	2	Three short-lived tremor episodes on 8 and 10/1/2020 with minor explosions.
1/21/2020 23:00	2020/03	2	Lava visible. High gas flux.
2/3/2020 20:00	2020/04	2	No further lava. Gas decreased but still elevated.
2/18/2020 23:00	2020/05	2	Steady decline in CO_2 and SO_2 but still elevated. Five lava lobes in vents. Water jetting in other vents.

Supplementary Table 2: Summary of scikit-learn classifier hyperparameter names and values checked in grid search. See scikit-learn documentation[1] for more information about parameter meanings.

**Classifier**	**Hyperparameter**	**Values**
Decision Tree	max_depth	3, 5, 7
	criterion	gini, entropy
	max_features	auto, sqrt, log2, None
Support Vector Machine	C	0.001, 0.01, 0.1, 1, 10
	kernel	poly, rbf, sigmoid
	degree	2,3,4,5
	decision_function_shape	ovo, ovr
k-Nearest Neighbours	n_neighbours	3,6,12,24
	weights	uniform, distance
	p	1,2,3
Random Forest	n_estimators	10, 30, 100
	max_depth	3, 5, 7
	criterion	gini, entropy
	max_features	auto, sqrt, log2, None
Neural Network	activation	identity, logistic, tanh, relu
	hidden_layer_sizes	10,100
Naive Bayes	var_smoothing	1.00E-09
Logistic Regression	penalty	l2, l1, elasticnet
	C	0.001, 0.01, 0.1, 1, 10

Supplementary References

1 Pedregosa, F. *et al.* Scikit-learn: Machine learning in Python. *Journal of machine learning research* **12**, 2825-2830 (2011).
